# Frailty and Its Associated Risk Factors: First Phase Analysis of Multicentre Indonesia Longitudinal Aging Study

**DOI:** 10.3389/fmed.2021.658580

**Published:** 2021-04-29

**Authors:** Siti Setiati, Czeresna Heriawan Soejono, Kuntjoro Harimurti, Noto Dwimartutie, I. G. P. Suka Aryana, Sri Sunarti, Fatichati Budiningsih, Roza Mulyana, Lazuardhi Dwipa, Agus Sudarso, Rensa Rensa, Rahmi Istanti, Muhammad Khifzhon Azwar, Jessica Marsigit

**Affiliations:** ^1^Division of Geriatrics, Department of Internal Medicine, Faculty of Medicine, Cipto Mangunkusumo Hospital, Universitas Indonesia, Jakarta, Indonesia; ^2^Clinical Epidemiology and Evidence-Based Medicine Unit, Faculty of Medicine, Cipto Mangunkusumo Hospital, Universitas Indonesia, Jakarta, Indonesia; ^3^Department of Internal Medicine, Faculty of Medicine, Universitas Udayana, Bali, Indonesia; ^4^Department of Internal Medicine, Faculty of Medicine, Universitas Brawijaya, Malang, Indonesia; ^5^Department of Internal Medicine, Faculty of Medicine, Universitas Sebelas Maret, Solo, Indonesia; ^6^Department of Internal Medicine, Faculty of Medicine, Universitas Andalas, Padang, Indonesia; ^7^Department of Internal Medicine, Faculty of Medicine, Universitas Padjajaran, Bandung, Indonesia; ^8^Department of Internal Medicine, Faculty of Medicine, Universitas Hasanuddin, Makasar, Indonesia; ^9^Department of Internal Medicine, Faculty of Medicine, Universitas Atma Jaya, Jakarta, Indonesia

**Keywords:** frailty, prevalence, Indonesia, community-dwelling elderly, long-term care, risk factors

## Abstract

**Background:** National long-term care development requires updated epidemiological data related to frailty. We aimed to find the prevalence of frailty and its associated factors among Indonesian elderly.

**Methods:** We conducted first-phase cross-sectional analysis of Indonesia Longitudinal Aging Study (INALAS) data collected from community-dwelling outpatients aged 60 years and older without acute illness in nine geriatric service care centres. Descriptive, bivariate and multivariate analyses were conducted.

**Results:** Among 908 elderly in this study, 15.10% were robust, 66.20% were pre-frail, and 18.70% were frail. Functional dependence was associated with frailty among Indonesian elderly (OR 5.97, 95% CI 4.04–8.80). Being depressed and at risk for malnutrition were also associated with frailty with OR 2.54, 95% CI 1.56–4.12, and OR 2.56, 95% CI 1.68–3.90, respectively. Prior history of fall (OR 1.77, 95% CI 1.16–2.72) and hospitalization (OR 1.46, 95% CI 0.97–2.20) in the previous 12 months were associated with frailty. There is also significant association between poly pharmacy and frailty (OR 2.42, 95% CI 1.50–3.91).

**Conclusion:** Approximately one in five Indonesian community-dwelling elderly was frail. Frailty is associated with functional dependence, being at risk for malnutrition or being malnourished, depression, history of fall, history of hospitalization, and poly pharmacy. There may be bidirectional relationships between the risk factors and frailty. The development of long-term care in Indonesia should be considered, without forcing the elderly who need it.

## Introduction

Indonesia is facing an increase in elderly population. Currently, there are more than 26 million people aged 60 years and older nationwide. This group of people contribute to 9.92% of total Indonesian population in 2020 (1). It is expected that elderly will make up 12.9% of total Indonesian population in 2030 (2). Frailty is a major problem among Indonesian elderly. This modern geriatric syndrome is closely related to high risk for devastating conditions, such as falls, hospitalization, disability, and death (3, 4).

Physiological decline of organs in the elderly and the comorbidities contribute to the change in functional status, depression state, cognitive function, and nutritional status, resulting in frail state. Frailty is a state of increased vulnerability to stressor resulting from a decline in physiological reserve and function capacity of an elderly. This vulnerable state may bring about inadequacy to recover after destabilization (5).

Frail older people are at higher risk of needing long-term care (LTC) (6). At the moment, Indonesia is in the process of developing LTC service for elderly population with significant loss of capacity. The funding of the development requires involvement of the policy makers, who make decisions based on the cost-efficiency and the urgency from the latest national prevalence of frailty. The data related to frailty are also essential for the development of other healthcare services that should be available in Indonesia to support World Health Organization (WHO) framework on healthy aging. However, the updated epidemiological data are lacking. Our previous multicentre cross-sectional study in 2014 suggested that frailty was found in 27.2% Indonesian elderly. Only 13.2% of Indonesian elderly were in robust condition (5).

We aimed to find the current prevalence of frailty and its associated factors among Indonesian community-dwelling elderly in this cross-sectional study. This first phase analysis is a part of the national multicentre data analyses of Indonesia Longitudinal Aging Study (INALAS).

## Materials and Methods

### Study Design and Subjects

This cross-sectional study was conducted in several geriatric service care centers in different islands of Indonesia from March to October 2020. In this study, we randomly selected 9 out of 17 elderly healthcare centers in Indonesia, namely Dr. Wahidin Sudirohusodo Hospital (RSWS), Makassar, South Sulawesi; Sanglah Hospital (RSUP Sanglah), Denpasar, Bali; Hasan Sadikin Hospital (RSHS) Bandung, West Java; Dr. M. Djamil Hospital (RSUP Dr. M. Djamil) Padang, West Sumatera; Dr. Moewardi Hospital (RSUD Moewardi), Solo, Central Java; Teja Husada Hospital (RSK Geriatri Teja Husada) and Ben Mari Hospital, Malang, East Java; Atma Jaya Hospital, and Cipto Mangunkusumo National General Hospital (RSCM), Jakarta. The data analysis was a part of INALAS. INALAS is a multicenter longitudinal aging study focusing on the health of older adults in Indonesia involving several geriatric service care centers in different islands. We planned to document the changes in a prospective manner every 6 months with target follow-up period of 5 years.

The inclusion criteria were community-dwelling elderly in the geriatric outpatient clinics of all selected hospitals who agreed to participate in the study. The definition of elderly was individuals aged 60 years and above. We recruited the patients consecutively. The exclusion criteria were elderly patients with an acute illness, such as acute confusional state, acute infection, and acute cerebrovascular and/or cardiovascular events.

The sample size needed for the study was determined based on the formula for the sample size of the estimated proportion (7). The minimum number of subjects to be recruited was 485 subjects. Ethical approval was obtained from the Faculty of Medicine, Universitas Indonesia. All subjects or their representing family members signed the written informed consent form.

### Data Collection

We used primary data from questionnaires and secondary data from medical records to collect the data related to the list of medications and comorbidities. Frailty state was assessed using the FRAIL scale, consisting of fatigue, resistance (defined as the ability to climb one flight of stairs), ambulation (walk a block), number of comorbid illnesses <5, and weight loss of more than 5% in the previous year. One point was given if the patients answered yes to each question. If the total score was 0, the patient was categorized as robust or fit. If the total score was 1 or 2, the patient was categorized as pre frail, and if the total score was 3 or higher, the patient was categorized as frail (8).

The data collected for the cross-sectional study included (a) demographic data (i.e., age, sex, last completed formal education, having caregiver or not, and marriage status); (b) History of fall and/or hospitalization in the past 12 months (c) frailty state based on the FRAIL questionnaire; (c) functional status based on the Barthel Index of Activity of Daily Living (ADL) questionnaire: totally dependent (score 0–4), severely dependent (score 5–8), moderately dependent (score 9–11), slightly dependent (score 12–19), independent (score 20); (d) nutritional status based on the Mini Nutritional Assessment Short-Form: normal nutritional status (score 12–14), at risk for malnutrition (score 8–11), malnourished (score 0–7); (e) depression status based on 15-item version of Geriatric Depression Scale: normal (score <5), depressed (score 5–15); (f) cognitive function based on Mini-Cog©: low likelihood of dementia (score 3–5) and high likelihood of dementia (score 0–2); (g) total number of the medications administered daily from self-report and/or medical record; (h) comorbidities obtained from medical record. All screening and diagnostic activities were done by physicians in internal medicine - geriatric medicine care centers.

### Statistical Analysis

The prevalence of frailty was obtained by calculating the proportion of patients who were categorized as frail divided by total study subjects. For the statistical analysis, frailty status was divided into: (1) non-frail (robust and pre frail), and (2) frail. Categories of age group were: (1) <70 years, and (2) ≥70 years. We determined the age groups based on the latest life expectancy of Indonesian population, approximately 70 years for both male and female individuals (1). The subjects were categorized based on their sex into male and female. Categories of the level of education were: (1) senior high school or higher, (2) junior high school or lower. Categories of the marriage status were: (1) married and (2) not married or widowed. Categories of the functional status were divided into two categories: (1) independent, (2) dependent (for subjects with total, severe, moderate and slight dependency). Categories of the depression status were: (1) normal and (2) depressed. Categories of cognitive function were: (1) low likelihood and (2) high likelihood of dementia. Categories according to nutritional status were: (1) normal and (2) at risk for malnutrition or malnourished (for subjects with score <12). Categories according the history of fall were: (1) no history of fall and (2) prior history of fall. Categories according the history of hospitalization were: (1) no history of hospitalization and (2) prior history of hospitalization. Poly pharmacy was determined based on the number of drugs that the patients consumed routinely and/or based on the medical records. An individual experiences poly pharmacy if he/she takes five or more medications daily (9).

Analyses were performed with SPSS Version 21 (IBM, Armonk, New York, USA). We used Chi-square test to perform the bivariate analysis, followed by multivariate analysis to assess the association between frailty and the independent variables. Variables with *p*-value < 0.25 in bivariate analysis were included for multivariate analysis using multiple logistic regression method. *P*-value < 0.05 was considered statistically significant.

## Results

**Figure 1:**
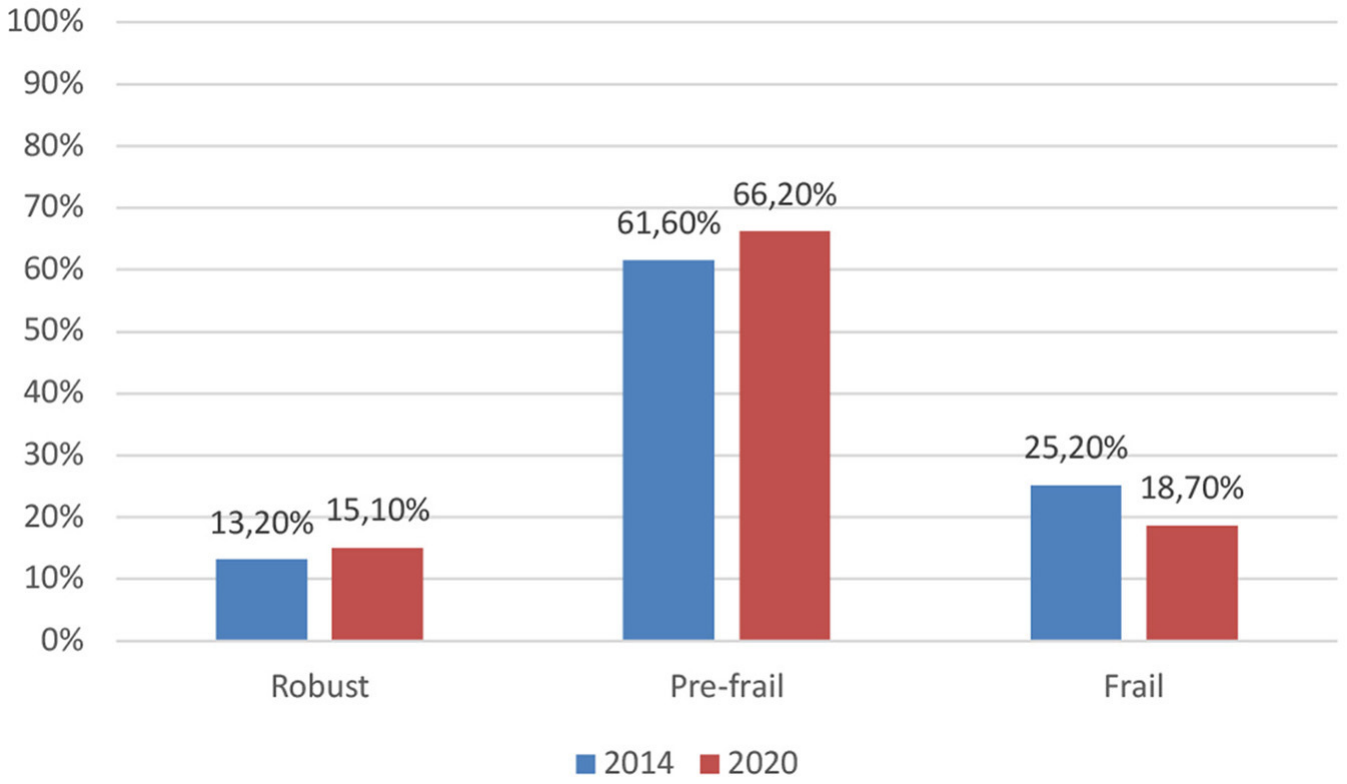
Prevalence of frailty among Indonesian elderly in 2014 and 2020.

We collected data from 908 individuals from different geriatric care centers. Male-to-female ratio of study subject was nearly 1:1. The characteristics of the subjects are shown in Table 1. Among the elderly in this study, 15.10% were robust, 66.20% were pre-frail, and 18.70% were frail, see Figure 1.

**Table 1:** Characteristics of Subjects (*n* = 908).

**Characteristics**	***n* (%)**
**Sex**	
Male	438 (48.2)
Female	470 (51.8)
**Age**	
60–69 years old	484 (53.3)
> 70 years old	424 (46.7)
**Educational background**	
Senior high school and higher	571 (62.9)
Junior high school and lower	337 (37.1)
**Marital status**	
Married	649 (71.5)
Not married/widowed	259 (28.5)
**Caregiver**	
Yes	531 (58.5)
No	377 (41.5)
**Functional status**	
Independent	667 (73.5)
Dependent	241 (26.5)
**Depression status**	
Normal	783 (86.2)
Depressed	125 (13.8)
**Nutritional status**	
Normal	644 (70.9)
At risk for malnutrition or malnourished	264 (29.1)
**Cognitive function**	
Low likelihood of dementia	806 (88.8)
High likelihood of dementia	102 (11.2)
**History of fall**	
No	726 (80.0)
Yes	182 (20.0)
**History of hospitalization**	
No	628 (69.2)
Yes	280 (30.8)
**Poly pharmacy**	
No	265 (29.2)
Yes	643 (70.8)
**Comorbidities**	
Hypertension	686 (75.6)
Diabetes mellitus	449 (49.4)
Coronary heart disease	295 (32.5)
Dyslipidaemia	396 (43.6)
Osteoarthritis	82 (9.0)
Chronic kidney disease	231 (25.4)
Visual impairment	78 (8.6)
Osteoporosis	16 (1.8)

Three out of five frail elderly were female, see Table 2. A higher proportion of frail elderly was aged 70 years and older (54.7%), completed senior high school and higher education (63.5%), was married (64.1%), and had caregiver (76.5%). More than 60% of frail subjects were functionally dependent. More than half of the frail elderly were also at risk for malnutrition or even were malnourished. On the other hand, a larger proportion of frail elderly was not depressed (68.2%), and had low likelihood of dementia (85.9%), no prior history of fall (65.9%), and no history of hospitalization (57.1%). Seventy-nine percent of frail elderly experienced poly pharmacy. The most common comorbidities of frail elderly were hypertension (70.0%), diabetes mellitus (52.4%), and coronary heart disease (43.5%). Based on the *p*-value of the results of bivariate analysis, all variables, except educational background, were included in the multivariate analysis.

**Table 2:** The results of bivariate analysis of study variables.

	**Frailty state**			
**Variables**	**Non-frail [n (%)]**	**Frail [n (%)]**	***P* Value**	**OR (95% CI)**
**Sex**				
Male	367 (49.7)	71 (41.8)	0.06	1
Female	371 (50.3)	99 (58.2)		1.38 (0.98–1.93)
**Age**				
60–69 years old	407 (55.1)	77 (45.3)	0.02	1
> 70 years old	331 (44.9)	93 (54.7)		1.48 (1.06–2.08)
**Educational background**				
Senior high school and higher	463 (62.7)	108 (63.5)	0.85	1
Junior high school and lower	275 (37.3)	62 (36.5)		0.97(0.68–1.237)
**Marital status**				
Married	540 (73.2)	109 (64.1)	0.02	1
Not married or widowed	198 (26.8)	61 (35.9)		0.66 (0.46–0.93)
**Functional status**				
Independent	605 (82.0)	62 (36.5)	<0.001	1
Dependent	133 (18.0)	108 (63.5)		7.92 (5.50–11.41)
**Depression status**				
Normal	667 (90.4)	116 (68.2)	<0.001	1
Depressed	71 (9.6)	54 (31.8)		4.37 (2.92–6.56)
**Nutritional status**				
Normal	560 (75.9)	84 (49.4)	<0.001	1
At risk for malnutrition or malnourished	178 (24.1)	86 (50.6)		3.22 (2.28–4.55)
**Cognitive function**				
Low likelihood of dementia	660 (89.4)	146 (85.9)	0.19	1
High likelihood of dementia	78 (10.6)	24 (14.1)		1.39 (0.85–2.27)
**History of fall**				
No	614 (83.2)	112 (65.9)	<0.001	1
Yes	124 (16.8)	58 (34.1)		2.56 (1.77–3.72)
**History of hospitalization**				
No	531 (72.0)	97 (57.1)	<0.001	1
Yes	207 (28.0)	73 (42.9)		1.93 (1.37–2.72)
**Poly pharmacy**				
No	229 (31.0)	36 (29.2)	0.01	1
Yes	509 (69.0)	134 (78.8)		1.68 (1.12–2.49)

The results of multivariate analysis showed that functional dependence was associated with frailty among Indonesian elderly [OR 5.97, 95% Confidence Interval (CI) 4.04–8.80], see Table 3. Being at risk for malnutrition or being malnourished was associated with frailty (OR 2.56, 95% CI 1.63–3.90). Depression was also associated with frailty with OR 2.54, 95% CI 1.56–4.12. Prior history of fall and hospitalization were also associated with frailty (OR of history of fall 1.77, 95% CI 1.16–2.72; OR of history of hospitalization 1.46, 95% CI 0.97–2.20). There is also significant association between poly pharmacy and frailty (OR 2.42, 95% CI 1.50–3.91).

**Table 3:** The risk factors associated with frailty in Indonesian elderly.

**Risk factors**	**Coefficient B**	**Standard error**	***P*-value**	**OR (95% CI)**
Functional dependence	1.79	0.20	<0.001	5.97 (4.04–8.798)
At risk for malnutrition or being malnourished	0.94	0.22	<0.001	2.56 (1.68–3.90)
Depression	0.93	0.25	<0.001	2.54 (1.56–4.12)
Prior history of fall	0.58	0.22	0.01	1.77 (1.16–2.72)
Prior history of hospitalization	0.38	0.21	0.06	1.46 (0.97–2.20)
Poly pharmacy	0.89	0.24	<0.001	2.42 (1.50–3.91)

## Discussion

Among Indonesian community-dwelling elderly in this study, 15.10% were robust, 66.20% were pre-frail, and 18.70% were frail. Frailty is significantly associated with functional dependence, being at risk for malnutrition or being malnourished, depression, history of falls, history of hospitalization, and poly pharmacy. In contrast, age 70 years and older, female sex, high likelihood of dementia, lower level of education, or marital status was not associated with frailty.

We decided to use FRAIL scale instead of FI-40 for clinical practice and research purposes during COVID-19 pandemic, to protect the community-dwelling elderly by minimizing the time spent in healthcare centres. The feasibility of FRAIL scale in clinical setting allows the use of the promising tool to facilitate the translation of clinical research into medical practice (10). Routine screening of adults aged 70 years and older was recommended in The Asia-Pacific clinical practice guideline for the management of frailty (11).

The prevalence of frailty among Indonesian elderly was 18.70% in 2020, which is slightly lower than the estimated prevalence in 2014 (5). The Percentage of pre-frail and robust elderly in 2020 were higher than in 2014 (66.20% prevalence of pre-frailty in 2020 vs. 61.60%; 15.10% prevalence of robust elderly in 2020 vs. 13.20%). Follow-ups of frailty status are mandatory and regularly updated prevalence of frailty are crucial, both of which are incorporated in INALAS. Should frailty prevalence become high, it will be a threat to the elderly, as well as an alarm to health care professionals, researchers, and policy makers (12). The health system must be prepared for the care of older people with frailty (13). Despite the reversibility of frailty to robust state, emphasis should be placed on the prevention, detection, and management of the risk factors associated with frailty. This is because annual mean healthcare cost of frail elderly is more than twice higher than the estimated cost for robust elderly (13).

The prevalence of frailty in Indonesian in 2020 is similar to the prevalence of frailty in East Coast of Peninsular Malaysia based on Fried's phenotype of frailty (18.30%) (14). The prevalence of frailty was 15.9% in Bangkok metropolitan area (15), and 13.9% in Northern Thailand (16). On the other hand, the prevalence of frailty in urban district setting in Malaysia was only 5.7% (17). Previous study in Singapore, a developed city-state in Southeast Asia, found that the prevalence of frailty and prefrailty islandwide was 6.2% and 37%, respectively (18). A recent systematic review and meta-analysis of 56 studies (not including Indonesian data) related to the prevalence of frailty in community-dwelling elderly in low-income and middle-income countries (LMICs) suggested that the pooled prevalence of pre frailty was 49.3% (95% CI 46.4%−52.2%, I^2^ = 97.5%) and frailty was 17.4% (95% CI 14.4%-20.7%, I^2^ = 99.2%) (19).

We can infer that despite the decreasing prevalence of frailty in Indonesia in 2020 compared to 2014, the prevalence is among the highest in Southeast Asia. The national prevalence of frailty in 2020 was also higher than the pooled prevalence of frailty in other LMICs. In addition, nearly half of Indonesian elderly with disability in 2020 relied on self-medication without proper outpatient treatment for their health problems. Six percent of elderly with disability did not even treat themselves (1). Therefore, development of LTC in Indonesia should be considered. Currently, Indonesia still relies on the accessible integrated service center for older adults (Posyandu Lansia) that involves volunteers to do regular medical check-up services in the community. It was suggested that the elderly care service should be culturally accepted by the service recipients. Cultural and religious values should be incorporated in the care service to increase the efficacy of the programme in Indonesia (20).

However, once LTC is available in Indonesia, physicians could not simply force the elderly in need to receive it. It requires informed consent from the patients who need and want the LTC. This is due to the culture of Indonesian people in terms of respecting and taking care of the elderly until death occurred. In general, filial piety is an important moral tenet in Asian countries. Asian children are expected to provide care for their aging parents (20). Among all Indonesian older adults in 2020, 39.10% lived in three-generation homes, 27.85% lived with other family members (not in a three-generation household), 20.51% lived with his/her spouse, and only 9.80% lived alone (1).

There are still other conditions supporting the development of LTC in Indonesia. Following the discharge from the isolation ward, several dependent older adults were still equipped with nasogastric tube and/or indwelling catheter. During COVID-19 pandemic, several home care teams and/or the family caregivers at home may not provide optimal continuous care after discharge. Not all caregivers at home were able to accompany elderly with functional dependence all the time. Living at home with family members do not guarantee the surveillance and good quality of care for dependent elderly. The sustainability of home-based care by family members in the future were also a concern in Thailand, accompanied by possible increase in out-migration of adult children. Decreasing fertility rates in Asian countries, including Indonesia, may signify fewer caregivers for the elderly in the future (20).

Indonesia is not the only Association of Southeast Asian Nations (ASEAN) Plus Three (APT) country being ill prepared to cope with the demand for LTC. The Philippines, Vietnam, Lao People's Democratic Republic, Cambodia, and Myanmar also have underdeveloped LTC system. Those countries also have gross domestic product (GDP) per capita lower than $20,000. Although the population is still relatively young compared with Western societies, those countries should establish a sustainable LTC system to meet the increasing demands of LTC amidst limited economic resources (20). LTC is essential to ensure a dignified late life, and to support behaviors to enhance intrinsic and functional capacity of the elderly. Expenditure on health systems on older populations should be seen as an investment, not a cost (21).

By involving more geriatric service care centres in the country and higher number of samples compared to previous national study in 2014 (5), we identified more risk factors associated with frailty, see Table 3. Based on the data in 2014, frailty in Indonesia is associated with age 70 years and older (OR 2.72, 95% CI 1.58–4.76), functional dependence (OR 2.89, 95% CI 1.79–4.67), and being at risk for malnutrition or malnourished (OR 3.75, 95% CI 2.29–6.13). Unlike the result of previous national data analysis, age 70 years and older was not associated with frailty in the current study. The findings related to functional dependence and nutritional status in the current study supported the previous Indonesian data.

Functional dependence based on Barthel Index of ADL is associated with nearly 7 times higher risk for frailty than control group. The risk is much higher than previously reported in previous national study. There is inverted association between ADL and frailty (5). A recent meta-analysis of 12 studies concluded that frail elderly were nearly three times more likely to develop or have worsened disabilities in ADL. In other words, frailty is considered as a precursor of disability (22).

Frailty and malnutrition are related syndromes (23), regardless of whether the former or the latter occurs first (24). Being at risk for malnutrition or malnourished is associated with 2-fold higher risk for frailty in this study. Another longitudinal aging study in Asia suggested a possible closed-loop cyclical association between the two. This may in turn lead to a combined malnutrition-frailty state (24). Malnutrition has major impact on the health and social care services. Similar to the burden of healthcare cost of frail elderly (13), community-dwelling individuals with malnutrition were reported to have healthcare costs that are double the cost for non-malnourished individuals (25).

In this study, depression is associated with nearly three times higher risk for frailty in elderly. A systematic review and meta-analysis of 24 studies suggested a reciprocal interaction between frailty and depression. Each condition may be a risk factor for the development of the other. In addition, there is an association between each condition and increased incidence and prevalence of the other (26). There are several potential explanations for the relationship between depression and physical frailty. Both syndromes have common pathophysiological mechanisms including hormonal changes in hypothalamic-pituitary-adrenal (HPA) axis, impaired HPA-axis response to stressful events, and elevated diurnal cortisol. Higher level of salivary cortisol level has been reported to be associated with frailty among community-dwelling female elderly. Moreover, severely depressed elderly may have inactivity, reduced physical activity, and medication non-compliance. Such lifestyle factors are related to frailty syndrome of older people (27). In addition, social isolation is related to depression in the elderly (26). Essential public health strategy in 2020 during the COVID-19 pandemic includes social distancing and stay-at-home measures (28). Since stay-at-home measures may increase social isolation (28), thus physicians and the community should also pay attention to the psychosocial health of the elderly.

There is an association between prior self-reported history of fall in the previous 12 months and 2-fold risk for frailty in our study. Older people who experienced falls had significantly more health problems than patients without falls. Besides, the risk of falling in elderly is also linked to frailty. One of the tools to use in hospital admission setting was “Identification of Seniors at Risk” (ISAR) screening, which is supplementary to vital sign measurement for the elderly to predict both falls and frailty (29). Not only is fall a significant risk factor for frailty, history of hospitalization in the previous 12 months is also associated with frailty. This finding is concordant with the results of previous systematic review and meta-analysis of 8 studies suggesting that elderly with frailty had the highest risk for hospitalization, followed by pre frail and robust elderly (30).

Poly pharmacy has been long known to be related to frailty. In this study, poly pharmacy is associated with 2-fold higher risk for frailty. A meta-analysis of 25 observational studies suggested a bidirectional relationship between poly pharmacy and frailty. The former appeared to be a major contributing factor for development of frailty (31). Poly pharmacy itself is related to the use of inappropriate drugs that are either contraindicated or pose a high risk to the elderly (32). Approximately 70% of Indonesian elderly in our study experienced poly pharmacy. Moreover, potentially inappropriate medication is highly prevalent among Indonesian elderly in primary healthcare centres (52.2%) (33). Therefore, appropriate reduction of poly pharmacy should be a strategy in preventing and managing frailty (31). Physicians should utilize prescribing tools, e.g., START/STOPP and Beers criteria, for appropriate medication reconciliation in elderly population (32).

To the best of our knowledge, this multicentre cross-sectional data analysis had the largest number of samples among other frailty studies in the region. Indonesia is a multiracial archipelago and our study population may represent the real population better by involving different centres and islands. Since transition between robust and frailty state is a dynamic process, we determined to follow up the changes in upcoming years to provide recent data related to geriatric medicine in Indonesia through INALAS for policy makers, guideline development, and physicians. We also suggested new risk factors associated with frailty in Indonesia, which were not analyzed in the previous multicentre study. The data analysis also included appropriate adjustment for possible confounders.

In conclusion, among Indonesian elderly in this study, 15.10% were robust, 66.20% were pre-frail, and 18.70% were frail. The development of LTC in Indonesia should be considered, accompanied by respecting the local cultural values without forcing the elderly who need it. Frailty is significantly associated with functional dependence, being at risk for malnutrition or being malnourished, depression, history of falls, history of hospitalization, and poly pharmacy. There may be bidirectional relationships between aforementioned risk factors and frailty. Detection and prevention of either one of the factors, including frailty itself, should be emphasized in clinical practice for better outcome of elderly patients and lower healthcare costs.

## Data Availability Statement

The raw data supporting the conclusions of this article will be made available by the authors, without undue reservation.

## Ethics Statement

Ethical approval was obtained from the Faculty of Medicine, Universitas Indonesia. The patients/participants provided their written informed consent to participate in this study.

## Author Contributions

SSe, CS, KH, ND, IA, SSu, FB, RM, LD, AS, RR, RI, MA, and JM contributed to development of study concept and design. SSe, CS, KH, ND, IA, SSu, FB, RM, LD, AS, RR, and RI contributed to Acquisition of data. SSe, RI, MA, and JM contributed to analysis and interpretation of data. SSe, CS, KH, ND, RI, MA, and JM contributed to drafting of the manuscript. All authors contributed to the article and approved the submitted version.

## Conflict of Interest

The authors declare that the research was conducted in the absence of any commercial or financial relationships that could be construed as a potential conflict of interest.

## References

[1] Subdirektorat Statistik Pendidikan dan Kesejahteraan Sosial. Statistik Penduduk Lanjut Usia 2020. 1st ed. Jakarta: Badan Pusat Statistik (2020).

[2] Indonesia KKR. Analisis Lansia di Indonesia. Jakarta: Kementerian Kesehatan Republik Indonesia. (2017) p. 7–8.

[3] Davinelli S Corbi G Scapagnini G. Frailty syndrome: a target for functional nutrients? Mech Aging Dev. (2021) 195:111441. 10.1016/j.mad.2021.111441 33539905

[4] Corbi G Cacciatore F Komici K Rengo G Vitale DF Furgi G. Inter-relationships between gender, frailty and 10-year survival in older Italian adults: an observational longitudinal study. Sci Rep. (2019) 9:18416. 10.1038/s41598-019-54897-2 31804552 PMC6895198

[5] Setiati S Laksmi PW Aryana IGPS Sunarti S Widajanti N Dwipa L. Frailty state among Indonesian elderly: prevalence, associated factors, and frailty state transition. BMC Geriatr. (2019) 19:182. 10.1186/s12877-019-1198-8 31269921 PMC6609407

[6] ChenSHondaTNarazakiKChenTKishimotoHKumagaiS. Physical frailty and risk of needing long-term care in community-dwelling older adults: a 6-year prospective study in Japan. J Nutr Health Aging. (2019) 23:856–61. 10.1007/s12603-019-1242-631641736

[7] Sastroasmoro S Ismael S. Dasar-dasar Metodologi Penelitian Klinis. 5th ed. St. Jakarta: Sagung Seto (2014).

[8] Morley JE Malmstrom TK Miller DK. A simple frailty questionnaire (FRAIL) predicts outcomes in middle aged African Americans. J Nutr Health Aging. (2012) 16:601–8. 10.1007/s12603-012-0084-2 22836700 PMC4515112

[9] Masnoon N Shakib S Kalisch-Ellett L Caughey GE. What is polypharmacy? A systematic review of definitions. BMC Geriatr. (2017) 17:230. 10.1186/s12877-017-0621-2 29017448 PMC5635569

[10] Kojima G. Quick and simple FRAIL scale predicts incident activities of daily living (ADL) and instrumental ADL (IADL) disabilities: a systematic review and meta-analysis. J Am Med Dir Assoc. (2018) 19:1063–8. 10.1016/j.jamda.2018.07.019 30206033

[11] Dent E Lien C Lim WS Wong WC Wong CH Ng TP. The asia-pacific clinical practice guidelines for the management of frailty. J Am Med Dir Assoc. (2017) 18:564–75. 10.1016/j.jamda.2017.04.018 28648901

[12] Morley JE Vellas B Abellan van Kan G Anker SD Bauer JM Bernabei R. Frailty consensus: a call to action. J Am Med Dir Assoc. (2013) 14:392–7. 10.1016/j.jamda.2013.03.022 23764209 PMC4084863

[13] Salinas-Rodríguez A Manrique-Espinoza B Heredia-Pi I Rivera-Almaraz A Ávila-Funes JA. Healthcare costs of frailty: implications for long-term care. J Am Med Dir Assoc. (2019) 20:102–3.e2. 10.1016/j.jamda.2018.09.019 30424982

[14] Mohd Hamidin FA Adznam SN Ibrahim Z Chan YM Abdul Aziz NH. Prevalence of frailty syndrome and its associated factors among community-dwelling elderly in East Coast of Peninsular Malaysia. SAGE Open Med. (2018) 6:1–11. 10.1177/2050312118775581 29872529 PMC5977425

[15] Wongtrakulruang P Muangpaisan W Panpradup B Tawatwattananun A Siribamrungwong M Tomongkon S. The prevalence of cognitive frailty and pre-frailty among older people in Bangkok metropolitan area: a multicenter study of hospital-based outpatient clinics. J Frailty, Sarcopenia Falls. (2020) 05:62–71. 10.22540/JFSF-05-062 32885103 PMC7461353

[16] Thinuan P Siviroj P Lerttrakarnnon P Lorga T. Prevalence and potential predictors of frailty among community-dwelling older persons in Northern Thailand: a cross-sectional study. Int J Environ Res Public Health. (2020) 17:4077. 10.3390/ijerph17114077 32521642 PMC7312471

[17] Sathasivam J Kamaruzzaman SB Hairi F Ng CW Chinna K. Frail elders in an urban district setting in Malaysia. Asia Pacific J Public Heal. (2015) 27(suppl. 8):52S−61S. 10.1177/1010539515583332 25902935

[18] Merchant RA Chen MZ Tan LWL Lim MY Ho HK van Dam RM. Singapore healthy older people everyday (HOPE) study: prevalence of frailty and associated factors in older adults. J Am Med Dir Assoc. (2017) 18:734.e9–734.e14. 10.1016/j.jamda.2017.04.020 28623152

[19] Siriwardhana DD Hardoon S Rait G Weerasinghe MC Walters KR. Prevalence of frailty and prefrailty among community-dwelling older adults in low-income and middle-income countries: a systematic review and meta-analysis. BMJ Open. (2018) 8:e018195. 10.1136/bmjopen-2017-018195 29496895 PMC5855322

[20] Yeung W-JJ Thang LL. Long-Term care for older adults in ASEAN plus three: the roles of family, community, and the state in addressing unmet eldercare needs. J Aging Health. (2018) 30:1499–515. 10.1177/0898264318796345 30239253

[21] World Health Organization. World Report on Aging and Health. Geneva: WHO Press (2015).

[22] Kojima G. Frailty as a predictor of disabilities among community-dwelling older people: a systematic review and meta-analysis. Disabil Rehabil. (2017) 39:1897–908. 10.1080/09638288.2016.1212282 27558741

[23] Roberts HC Lim SER Cox NJ Ibrahim K. The challenge of managing undernutrition in older people with frailty. Nutrients. (2019) 11:808. 10.3390/nu11040808 30974825 PMC6521101

[24] Wei K Nyunt M-S-Z Gao Q Wee S-L Yap K-B Ng T-P. Association of frailty and malnutrition with long-term functional and mortality outcomes among community-dwelling older adults. JAMA Netw Open. (2018) 1:e180650. 10.1001/jamanetworkopen.2018.0650 30646023 PMC6324309

[25] Guest JF Panca M Baeyens J-P de Man F Ljungqvist O Pichard C. Health economic impact of managing patients following a community-based diagnosis of malnutrition in the UK. Clin Nutr. (2011) 30:422–9. 10.1016/j.clnu.2011.02.002 21406315

[26] Soysal P Veronese N Thompson T Kahl KG Fernandes BS Prina AM. Relationship between depression and frailty in older adults: a systematic review and meta-analysis. Aging Res Rev. (2017) 36:78–87. 10.1016/j.arr.2017.03.005 28366616

[27] Buigues C Padilla-Sánchez C Garrido JF Navarro-Martínez R Ruiz-Ros V Cauli O. The relationship between depression and frailty syndrome: a systematic review. Aging Ment Health. (2015) 19:762–72. 10.1080/13607863.2014.967174 25319638

[28] Office EE Rodenstein MS Merchant TS Pendergrast TR Lindquist LA. Reducing social isolation of seniors during COVID-19 through medical student telephone contact. J Am Med Dir Assoc. (2020) 21:948–50. 10.1016/j.jamda.2020.06.003 32674825 PMC7274632

[29] Song X Mitnitski A Rockwood K. Prevalence and 10-year outcomes of frailty in older adults in relation to deficit accumulation. J Am Geriatr Soc. (2010) 58:681–7. 10.1111/j.1532-5415.2010.02764.x 20345864

[30] Chang S-F Lin H-C Cheng C-L. The relationship of frailty and hospitalization among older people: evidence from a meta-analysis. J Nurs Scholarsh. (2018) 50:383–91. 10.1111/jnu.12397 29874399

[31] Gutiérrez-Valencia M Izquierdo M Cesari M Casas-Herrero Á Inzitari M Martínez-Velilla N. The relationship between frailty and polypharmacy in older people: a systematic review. Br J Clin Pharmacol. (2018) 84:1432–44. 10.1111/bcp.13590 29575094 PMC6005607

[32] Nwadiugwu MC. Frailty and the risk of polypharmacy in the older person: enabling and preventative approaches. J Aging Res. (2020) 2020:1–6. 10.1155/2020/6759521 32676209 PMC7341397

[33] Abdulah R Insani WN Destiani D Rohmaniasari N Mohenathas N Barliana MI. Polypharmacy leads to increased prevalence of potentially inappropriate medication in the Indonesian geriatric population visiting primary care facilities. Ther Clin Risk Manag. (2018) 14:1591–7. 10.2147/TCRM.S170475 30233194 PMC6129028

